# Discovery of differentially expressed lncRNAs in porcine ovaries with smaller and bigger litter size

**DOI:** 10.3389/fgene.2025.1498076

**Published:** 2025-04-16

**Authors:** Saixing Duan, Falei Li, Libing Meng, Shimei Cheng, Huangqi Shi, Yingjie Qu, Chaoyue He, Shengyu Gao, Jian Li, Shiduo Sun, Yong Liu, Gaoxiao Xu

**Affiliations:** ^1^ Anhui Province Key Laboratory of Embryo Development and Reproductive Regulation, Anhui Province Key Laboratory of Environmental Hormone and Reproduction, School of Biological and Food Engineering, Fuyang Normal University, Fuyang, China; ^2^ Shaanxi Key Laboratory of Molecular Biology for Agriculture, College of Animal Science and Technology, Northwest A&F University, Yangling, Shaanxi, China

**Keywords:** lncRNA, pig, ovary, litter size, ceRNA

## Abstract

**Introduction:**

The number of litters is an important reproductive trait, which is one of the main indicators reflecting the production level and economic benefit of the pig farm. As an important reproductive organ of female mammals, the ovary is controlled by a complex transcription network of coding and non-coding genes to undergo a series of biological processes during each estrus cycle, thereby regulating the reproductive capacity of the sow. However, these molecular regulation mechanisms affecting sow litter size are still unclear.

**Methods:**

Regarding the non-coding molecular regulatory mechanisms in ovarian function with smaller and larger litter size (SLS and LLS), we investigated the expression profile of lncRNA in pig SLS and LLS samples. Total RNAs from porcine ovaries were used to construct libraries using Ribo-Zero RNA sequencing method.

**Results:**

Here we profiled the expression of lncRNA in porcine ovaries with SLS and LLS, and identified a total of 3,556 lncRNA candidates, of which 96 were upregulated lncRNA and 206 were downregulated lncRNA when comparing LLS to SLS. In addition, a competitive endogenous RNA (ceRNA) network was constructed, and it was found that lncRNAs LOC100513133 and LOC102168075 may serve as ceRNAs containing potential binding sites for miR-26b, let-7g and miR-125b.

**Discussion:**

These results demonstrate that lncRNAs may play roles in modulating porcine litter size.

## Introduction

The number of litters is an important reproductive trait and one of the main indicators reflecting the production level and economic benefits of pig farms (Vázquez-Gómez et al., 2020; Zheng et al., 2020). The increasing number of litters and shorter delivery intervals to obtain economic benefits has always been a global goal for sow feeding (Hidalgo et al., 2020; Schneider et al., 2015). As an important reproductive organ of female mammals, the ovary is controlled by a complex transcription network of coding and non-coding genes (Pan et al., 2014; Yin et al., 2020; Du et al., 2019).

Non-coding RNA, for example, long non-coding RNAs (lncRNAs) are involved in ovarian function regulatory network and are important competing endogenous RNAs (ce RNA) (Zhang et al., 2018; Chen, 2016). LncRNA plays an indispensable role in the process of transcription and post-transcriptional regulation of gene activity through cis and trans regulation (Liao et al., 2020; Liu B. et al., 2020; Meng et al., 2020), such as functioning as a molecular scaffold in the assembly of transcription complexes (Ponzio et al., 2017; Hou et al., 2017), or regulating RNA-protein interactions and alternative splicing (Liu S. et al., 2020; Meng et al., 2020), or acting as a microRNA (miRNA) sponge and relieving miRNA inhibition of post-transcriptional expression of their target genes (Wei et al., 2018). In the nucleus, X-inactive-specific transcript (Xist) is strictly localized within chromosome boundary and triggers chromosome remodeling to achieve stable silencing with relatively few genes remaining active (Zhang et al., 2019). Non-coding RNA SRA was found to be immunoprecipitated with MyoD, thereby affecting muscle differentiation (Hubé et al., 2011). In the cytoplasm, lncMD had been demonstrated to function as competing endogenous RNAs (ceRNAs) to bind miRNAs and regulate cattle myoblast differentiation (Sun et al., 2016).

With the rapid development of high-throughput sequencing technology and bioinformatics, lncRNAs have been found in various tissues of different species, and the dynamic expression patterns of lncRNAs under different developmental stages and physiological conditions have been revealed. They emerge as key regulators of major biological processes impacting proliferation, differentiation, and apoptosis in every branch of life, including ovarian function (Wang et al., 2019). Anna found that some differentially expressed lncRNAs were involved in spermatogenesis in pig testis tissues by transcriptome analysis (Esteve-Codina et al., 2011). Ran performed RNA sequencing (RNA-seq) analysis of pig mature and immature testicular tissues (Ran et al., 2016). Wang performed RNA-seq on endometrial tissues of pigs during pregnancy and non-pregnancy, and identified a total of 34 lncRNAs with significant differences in expression (Wang et al., 2017). In this study, we characterized lncRNA expression profiles in six sow ovaries with smaller and larger litter size (SLS and LLS) using Ribo-Zero RNA-seq method combined with bioinformatics tools, thus uncovered novel molecules in the regulatory network of swine reproduction.

## Materials and methods

### Sample preparation

In China, PRRS and ring disease are one of the most important factors affecting litter size. Our experiment is to guide the pig breeding industry, so we chose sows without these two diseases. In addition, the sows we selected are of the same age, body condition, and feed with the same nutritional level. Most importantly, they have no history of reproductive diseases. We collected the litter size records of the Dabai Landrace binary cross sows from the Dahua breeding farm (Guangxi Hanshiwei Food Co., Ltd., from 2016 to 2018, a total of 8,557 litters), and used SPSS25.0 to perform a significance test on these data. After normal distribution conversion and inspection, it is found that the total litter size (12.9 ± 2.17) approximately obeys the normal distribution. The critical value of 15% right tail probability is 14.7 heads per litter, and the critical value of 15% left tail probability is 9.3 heads per litter. Ovarian tissues of pig under follicles of the same diameter were taken when static reflex occurred in pigs of the same parity.

Animal welfare and research plan were approved by Animal Care Committee of the Guangxi University. The ovaries of sows (Large White × Danish Landrace binary hybrid pig) were collected from a commercial pig farm (Nanning, Guangxi, China) 4 days after the fourth delivery. Six ovary tissues were chosen to perform RNA-Seq from pig with small (SLS, 8.48 ± 0.53/litter) and large (LLS, 16.19 ± 0.43/litter) litter size after the fourth delivery, respectively. The sample was were obtained and snap-frozen in liquid nitrogen.

### Library preparation and illumina sequencing

We subjected the extracted RNA from tissue samples of six pig ovaries (three pig ovaries from pigs with small litter size; three pig ovaries from pigs with large litter size) to agarose gel electrophoresis, and then RNA concentrations were quantified using Agilent 2100 Bioanalyzer and NanoDrop spectrophotometer. Ribosomal RNA was removed using Ribo-Zero™ rRNA Removal Kit (Epicentre, Madison, WI, United States). Library preparation and sequencing analysis were described as previous study (Shen et al., 2019). The raw data files were cleaned up for quality control through Trim Galore and then mapped to the pig reference genome (Sscrofa 11.1) from UCSC (Kim et al., 2013). CuffLinks were used to linear transcripts assembly and abundance assessment, then we identified reads from fusion transcripts that did not match the linear RNA sequence. The lncRNAs ≤100 kb in length, with at least two support sequences, and sequences with no more than two mismatches were retained for further analysis.

### Gene ontology and pathway analysis

Gene Ontology analysis (GO; http://www.geneontology.org) was used to explore potential functions of differentially expressed mRNA and lncRNA. GO terminology analyzes information about biological processes involving genes, and cellular components or metabolic pathways, and highlights molecular functions. In addition, we also performed the Kyoto Encyclopedia of Genes and Genomes pathway analysis (KEGG; http://www.kegg.jp), using DAVID to gain insight into the interaction and reaction network of differentially expressed lncRNA molecules. The -log_10_ P value represents a significant enrichment of a given GO term or KEGG pathway between upregulated and downregulated entities.

### CeRNA network construction of lnc RNA

Bnd lncRNA sequences, an interaction regulation network was constructed between mRNA-miRNA-lncRNA. The RNAhybrid (https://bibiserv.cebitec.uni-bielefeld.de/rnahybrid/) and TargetScan (http://www.targetscan.org/) were used to predict the interactions of miRNA-mRNA and miRNA-circRNA. The correlation between the expression levels of lncRNA and miRNA was calculated with SPSS Pearson correlation assay.

### CDNA synthesis, and real-time qPCR of lnc RNA

Invitrogen Trizol reagent (Thermo, MA, United States) was used to extract total RNA from porcine ovarian tissue, and PrimeScript™ RT kit (TaKaRa, Dalian, China) with gDNA eraser was used to reverse transcription of total RNA while removing genomic DNA. Real-time quantitative PCR (qPCR; 95°C 30 s, 95°C 5 s, 60°C 30 s for 40 cycles, following 70°C 10 min for elongation) of lnc RNA was performed using SYBR Green kit (TaKaRa, Dalian, China) in triplicate on a Bio-Rad CF96 system (Bio-Rad, United States), and the data was normalized using β-actin. The primers are listed in Supplementary Table S1.

### Statistical analyses

The data showed mean ± SEM. P-values were calculated by a Student’s t-test. P < 0.05 was considered as significant difference.

## Results

### Ribo-zero RNA-Seq of pig ovary

**FIGURE 1:**
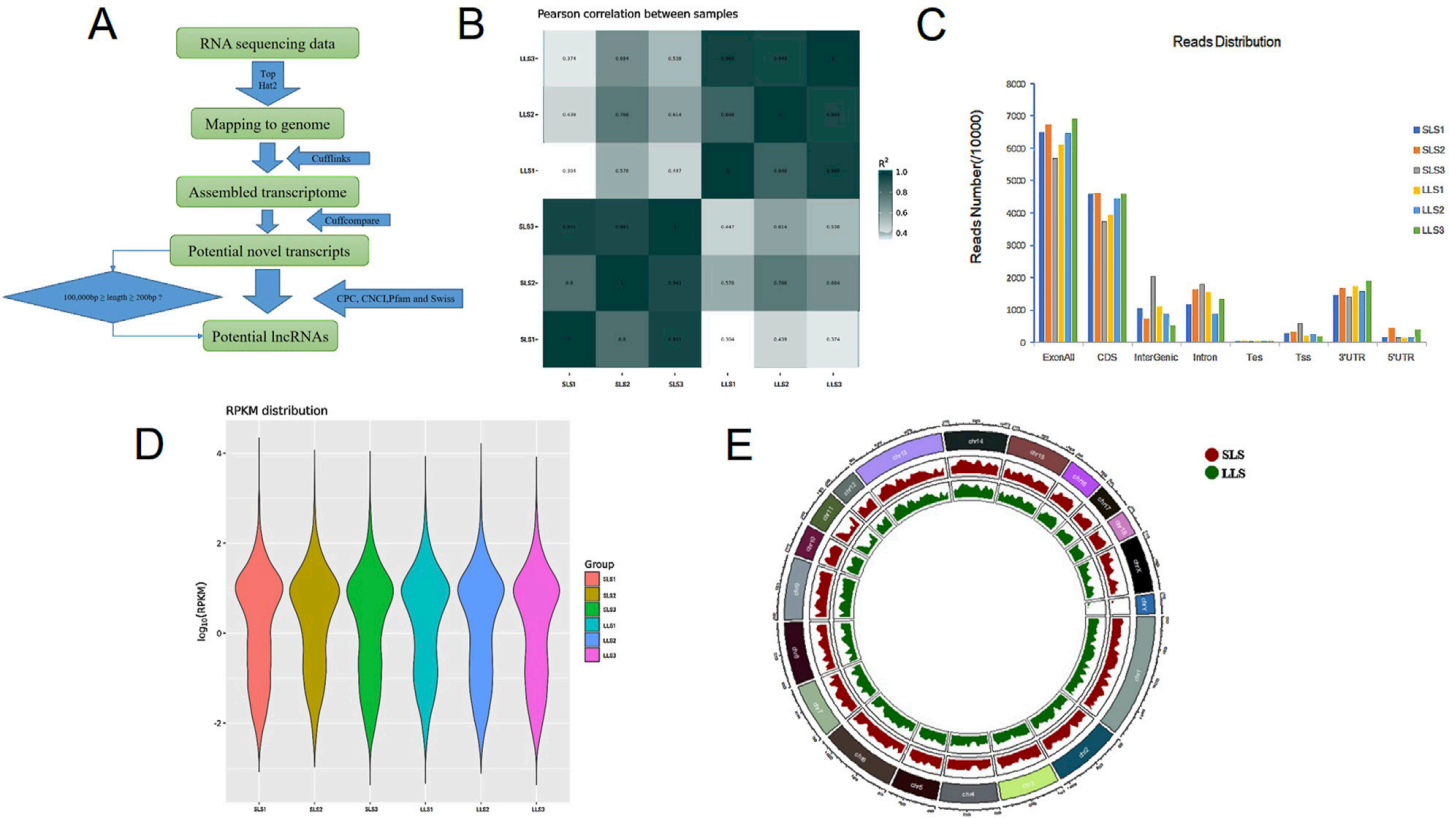
Analysis of ovarian transcriptome sequencing in large and small litter size pigs. **(A)** Identification process of lncRNAs in this study. **(B)** Principal component analysis (PCA) was used to analyze the pearson correlation between small litter size (SLS) and large litter size (LLS) of transcriptome data, and the two groups could be distinguished. n = 3. **(C)** The distribution of reads data in different regions of the genome. The SLS and LLS mean small litter size and large litter size, respectively. **(D)** The distribution of RNA expression (RPKM) in each sample. The SLS and LLS mean small litter size and large litter size, respectively. **(E)** The distribution of reads data on each chromosome in pigs. Red and green represent chromosomes of small litter size and large litter size of pig, respectively.

In this study, we selected six ovarian samples to perform Ribo-Zero RNA-Seq from pig with small (SLS, 8.48 ± 0.53/litter) and large (LLS, 16.19 ± 0.43/litter) litter size after the fourth delivery, respectively (Supplementary Table S2). The significance of large litter size was higher than that of small litter size (P < 0.001). We used Pfam and Cpat databases to filter, and analyzed the protein coding ability predictions of new lncRNAs through CPC and CNCI tools, and identified 3,556 potential lncRNA transcripts (Figure 1A; Supplementary Table S3). As shown in Figure 1B, Pearson correlation analysis showed that there is a good correlation between SLS and LLS ovarian samples. We obtained 75–83 and 78–85 million unique mapped clean reads from the SLS and LLS sample libraries (Table 1), and found that 69.7% of the reads are located in the exon and sequence coding for aminoacids in protein (CDS) regions, while the distribution in the non-coding (intergenic, intronic, UTR, transcription initiation site (TSS), and transcription end site (TES) regions markedly reduced (30.3%; Figure 1C).

**TABLE 1:** RNA-seq data mapping results statistics.

Item	All clean date reads	UnMapped reads	Mapped reads	Mapped rate	Unique mapped reads	Unique mapped rate
SLS1	83,705,572	3,284,502	80,421,070	96.08%	75,087,790	89.70%
SLS2	87,281,814	3,309,853	83,971,961	96.21%	79,268,408	90.82%
SLS3	91,009,668	3,344,482	87,665,186	96.33%	83,353,517	91.59%
LLS1	96,428,826	5,734,323	90,694,503	94.05%	80,723,407	83.71%
LLS2	88,161,908	4,054,317	84,107,591	95.40%	78,223,478	88.73%
LLS3	95,188,516	4,025,678	91,162,838	95.77%	85,574,633	89.90%

As shown in Figure 1D, the average expression level of the identified RNA including lncRNA and mRNA is 12.97 (FPKM), showing a lower expression level. In addition, we found that every chromosome could produce lncRNA, and the longer the chromosome, the more likely it is to produce lncRNA (Figure 1E).

### Differentially expressed mRNAs

**FIGURE 2:**
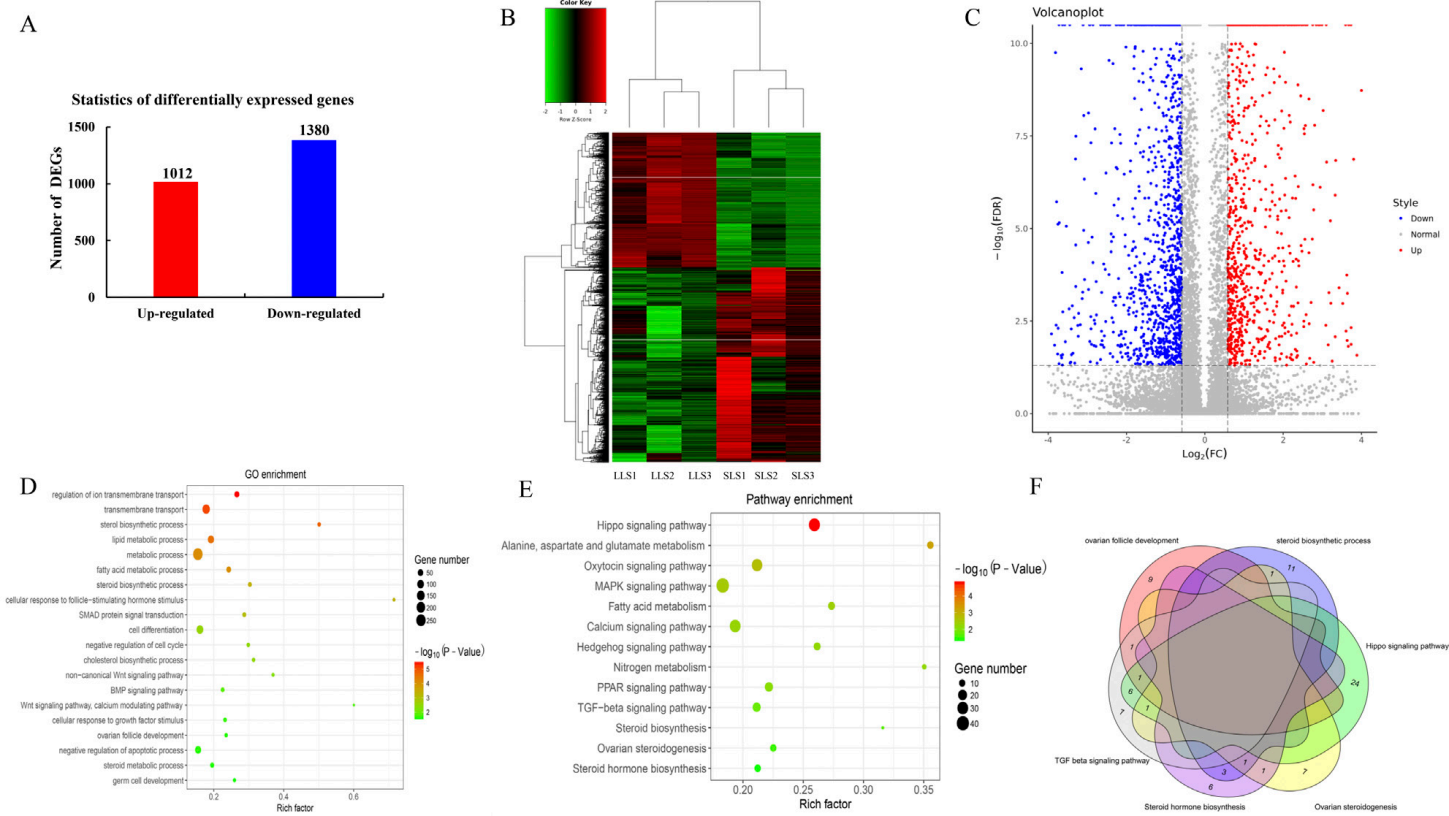
Analysis of differentially expressed genes and functional enrichment in ovarian transcriptome. **(A)** There were 1,012 upregulated genes and 1,380 downregulated genes in differentially expressed genes. **(B)** Heat map of differentially expressed genes between small litter size and large litter size. DEG expression levels are represented as FPKM-normalized log2-transformed counts. **(C)** Volcano map of differentially expressed genes, where red dots indicate upregulated genes, blue dots indicate downregulated genes, and gray dots indicate genes with no significant difference. **(D, E)** GO and KEGG signal pathway enrichment analysis of differentially expressed genes. The size of the dots represents the number of genes enriched, and the color of the dots represents the P value. GO, Gene Ontology; KEGG, Kyoto Encyclopedia of Genes and Genomes. **(F)** GO and KEGG analyses were combined and shown as Venn diagram. GO, Gene Ontology; KEGG, Kyoto Encyclopedia of Genes and Genomes.

In this study, 16,428 genes were detected (Supplementary Table S4), and 2,392 unigenes were significantly differently expressed at the LLS and SLS ovarian samples (P < 0.05; Figure 2A); all differentially expressed genes (DEGs) were shown in Supplementary Table S5. In order to verify the accuracy of RNA-seq, we randomly selected 8 genes (4 upregulated genes and 4 downregulated genes) for qPCR detection, and compared the qPCR data with the sequencing data (Table 2). It can be found that the qPCR results of the selected DEGs are basically similar to the RNA-seq results, indicating that the sequencing data has high accuracy.

**TABLE 2:** qPCR validation of DEGs between ovaries of SLS and LLS.

Gene name	Gene ID	RNA-Seq (log2FC)	qPCR validation (log2FC)
EPCAM	403163	4.712362912	4.412441190
ERO1β	100154974	4.514190014	4.183180192
LOC100525099	100525099	6.27660683	5.114782718
HSD17β2	100312973	5.816103904	1.394357375
LRP8	100187577	−1.902838053	−1.416831457
LRP2	88161908	−2.974581816	−2.145352780
ACTG2	100520667	−1.825145943	−2.709551033
SLIT1	100627880	−4.406230157	−3.586271999

As shown in Figure 2B, the cluster heat map of DEGs and the Pearson correlation in each sample were displayed. To further explore the potential functions of DEGs, we used volcano plots to compare their transcript expression abundance in SLS and LLS ovarian samples (Figure 2C).

To explore the possible biological functions of DEGs identified in SLS and LLS porcine ovarian tissues, GO analysis was performed (Figure 2D). The results showed that these DEGs were significantly enriched by 20 biological processes, including steroid biosynthesis process, lipid metabolism process, ovarian follicle development, granular cell differentiation, negative regulation of apoptosis process, and cell response to follicle stimulating hormone stimulation.

In order to better understand the molecular regulation of DEGs, we performed KEGG pathway analysis (Figure 2E). It showed that a total of 50 pathways are enriched, among which the most significant is the hippo signaling pathway (path: 04390), with 32 annotation genes. From the three enrichment pathways of steroid biosynthesis (path: 00100), ovarian steroid production (path: 04913) and steroid hormone biosynthesis (path: 00140), it could be seen that the synthesis and metabolism of steroids play an important role in the ovaries of pigs. In order to accurately obtain the key genes that may affect the pig litter size, we combined GO analysis and KEGG pathway analysis. The results showed that 15 genes may be candidate genes affecting piglet size traits. Among them, INHBA, BMP4 are related to ovarian follicle development, CAPN5 and FOXL2 are related to the regulation of granulosa cell differentiation; CYP21A2, STAR, HSD17B2, HSD17B8 and HSD17B12 are involved in steroid biosynthesis; In addition, the key genes TGFBR1 and TGFB3 in TGF signaling pathway are also significantly different (Figure 2F). It is worth noting that TGF signaling pathway is highly related to the regulation of reproductive performance.

### Differentially expressed lncRNAs

**FIGURE 3:**
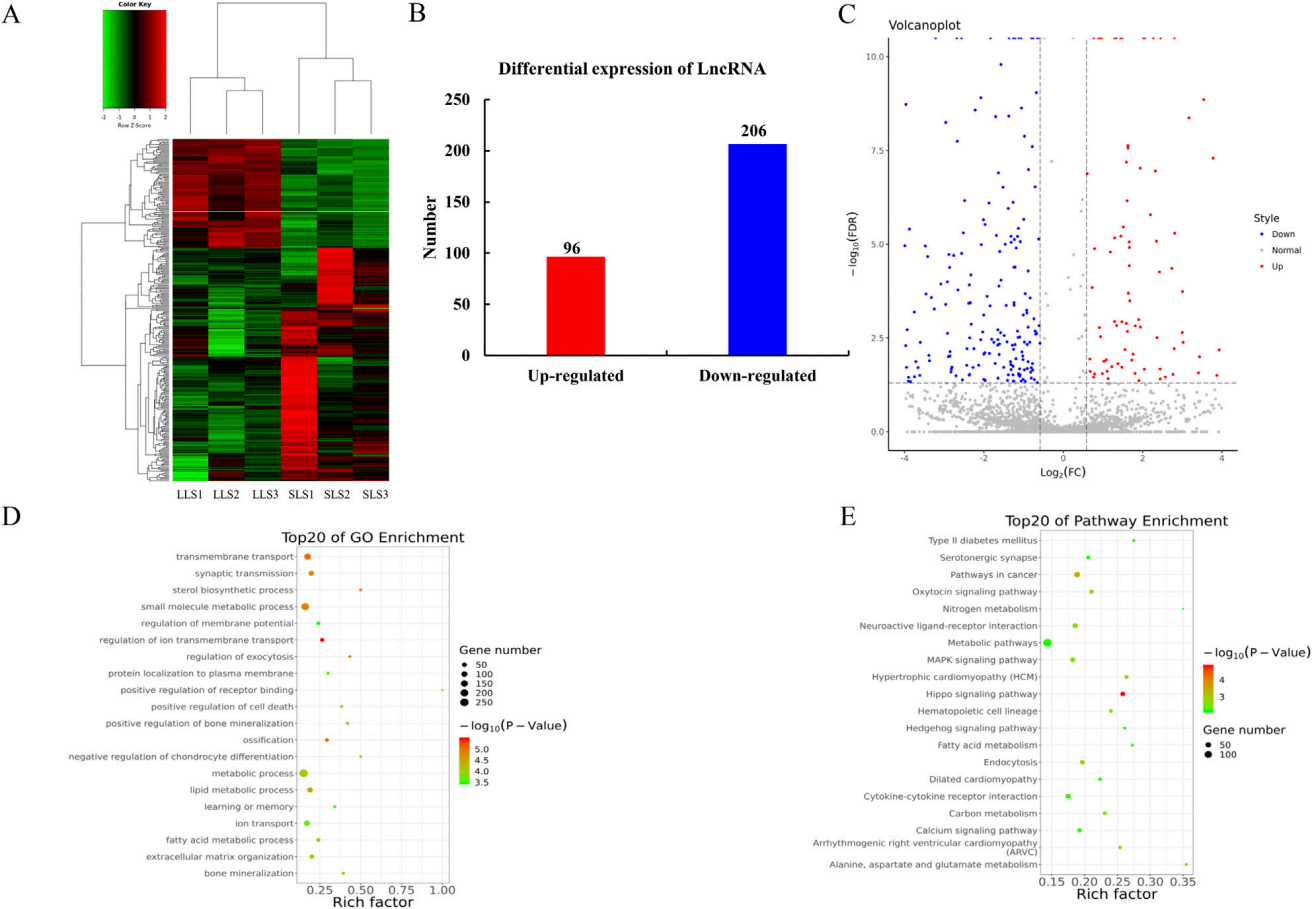
Differential expression and enrichment analysis of lncRNAs in ovarian transcriptome. **(A)** A heat map was drawn for 302 differentially expressed lncRNAs. DEG expression levels are represented as FPKM-normalized log2-transformed counts. **(B)** Among 302 differentially expressed lncRNAs, 97 were upregulated and 205 were downregulated. The SLS and LLS mean small litter size and large litter size, respectively. **(C)** In the volcano map of lncRNAs, red dots indicate upregulated lncRNAs, blue dots indicate downregulated lncRNAs, and gray dots indicate lncRNAs with no significant difference. **(D, E)** Go and KEGG enrichment analysis of differentially expressed lncRNAs target genes was performed. The results showed that the top 20 were significantly enriched. GO, Gene Ontology; KEGG, Kyoto Encyclopedia of Genes and Genomes.

From the fold change and the adjusted level of significance, a total of 302 differentially expressed lncRNAs were screened (P < 0.05; Figure 3A; Supplementary Table S6). We found that 206 lncRNAs were downregulated and 96 lncRNAs were upregulated (Figure 3B), and LLS group (FPKM = 346.38) had an obvious trend of high expression of lncRNA when comparing SLS group (FPKM = 256.71). It is worth mentioning that ALAS1 (FPKM = 32,089.67) and LOC100513133 (FPKM = 10,768) have the highest expression levels among all upregulated and downregulated lncRNAs, respectively. In order to better understand the expression changes and potential functions of lncRNA, a volcano plot is shown in Figure 3C.

LncRNA regulates the transcription and translation of coding genes in cis and trans regulation: when the role of lncRNA is limited to the same chromosome (for example, acting as a molecular scaffold), it performs cis regulation; when it affects gene expression on other chromosomes, trans regulation plays a role (for example, acting as a miRNA sponge). As shown in Figures 3D, E, the first 20 enriched GO terms and KEGG pathways are shown. Interestingly, metabolic processes in both GO and KEGG analyses had the most cis and trans’ target gene clusters, indicating that metabolic processes may play an important role in ovarian function.

**FIGURE 4:**
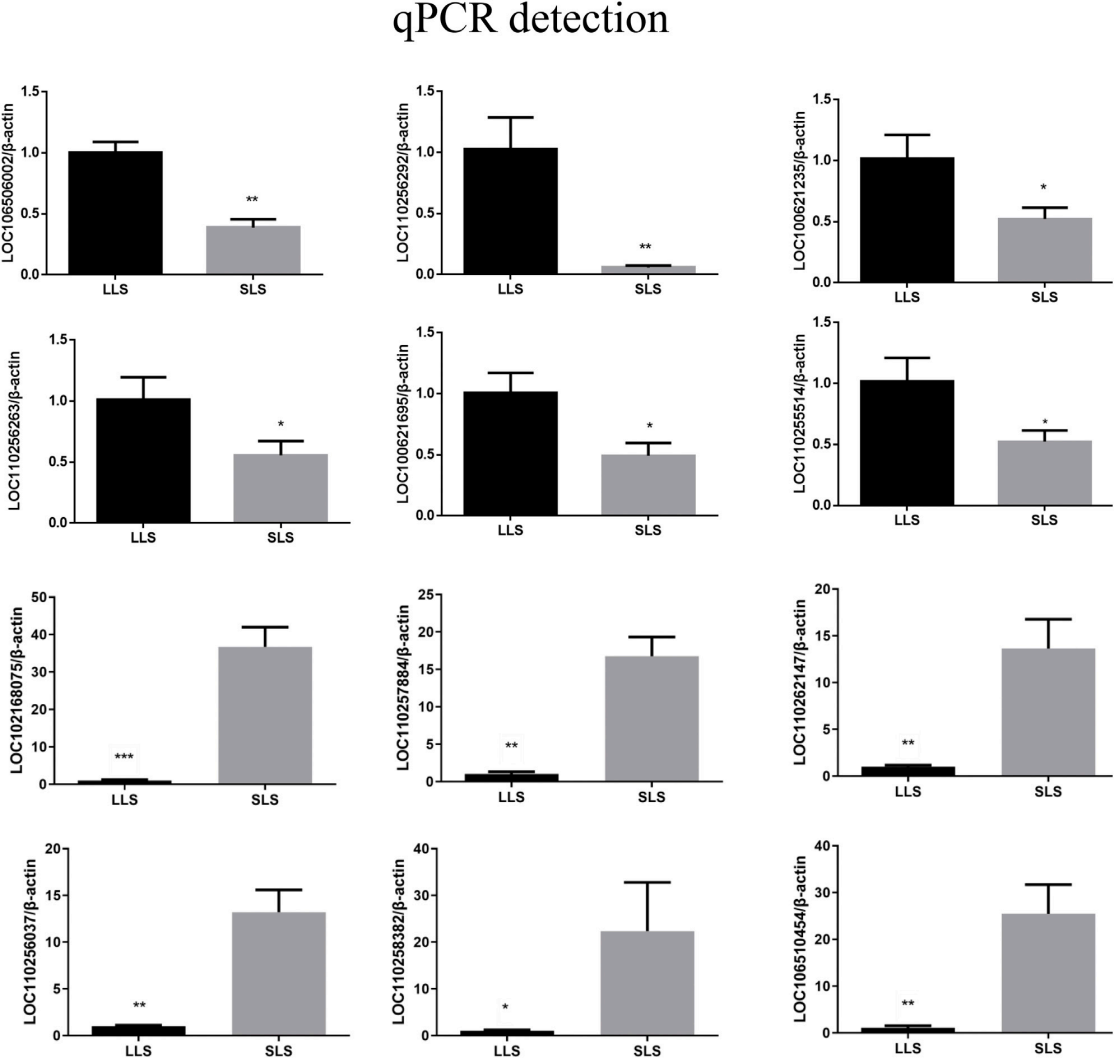
The expression level of lncRNAs was verified by qPCR assay. The transcriptomes differentially expressed lncRNAs, which were verified by qPCR. The internal reference gene was β-actin, with three biological replicates in each group. The SLS and LLS mean small litter size and large litter size, respectively.

In order to verify the accuracy of sequencing data, we randomly selected 12 genes (6 upregulated lncRNAs and 6 downregulated lncRNAs) for qPCR detection, and compared the qPCR data with the sequencing data (Figure 4; Supplementary Table S7). It can be found that the qPCR results of the selected differentially expressed lncRNAs are basically similar to the RNA-seq results, indicating that the lncRNA-Seq data are highly accuracy.

### CeRNAs networks

lncRNA can act as a molecular sponge of miRNA to sequester miRNA from its target gene, that is, miRNA is the common target of lncRNA and mRNA. We selected 9 miRNAs, 9 lncRNAs and 342 mRNAs related to ovarian development to construct an mRNA-miRNA-lncRNA (ceRNA) network (Supplementary Figure S1). For example, LOC100513133 and LOC102168075 have multiple binding sites for microRNAs related to ovarian development, such as miR-26b (Liu et al., 2016; Lin et al., 2012), let-7g (Zhou et al., 2016; Cao et al., 2015a) and miR-125b (Zhang D. et al., 2020; Yao et al., 2018; Yao et al., 2019). This ceRNA network may provide valuable information for ovarian function.

## Discussion

The next-generation sequencing shows that endogenous lncRNA is usually expressed in a variety of pig tissues, including ovaries, in a spatio-temporal specific manner. It was found that some differentially expressed lncRNAs were involved in spermatogenesis in pig testis tissues by transcriptome analysis. In addition, during pregnancy and non-pregnancy, a total of 34 lncRNAs were differentially expressed in pig endometrial tissues and involved in various biological processes (Wang et al., 2017). In this study, 16,428 genes were detected, and 2,392 genes were significantly differently expressed at the SLS and LLS ovarian samples. GO and KEGG analysis showed that 15 genes may be candidate genes affecting piglet size traits. Moreover, 302 lncRNAs were significantly differently expressed in SLS and LLS ovarian samples. ALAS1 and LOC100513133 showed the highest expression level of all upregulated and downregulated lncRNAs, revealing that they may has the potential to perform important functions on modulating porcine litter size.

Studies have shown that LncRNA regulates gene expression during the growth and development of different tissues in mammals, including ovaries. According to its position in the cell, lncRNA usually has a variety of regulatory mechanisms. In the cell nucleus, lncRNA can act as a molecular scaffold to recruit transcriptase or transcription factors to mediate the transcription of coding genes. In the cytoplasm, lncRNA acts as a ceRNA to competitively bind to miRNA, thereby reducing the inhibitory effect of miRNA on target genes (Sun, et al., 2016). As an important reproductive organ of female mammals, the ovary is controlled by a complex transcription network of coding and non-coding genes to undergo a series of biological processes during each estrus cycle, thereby regulating the reproductive capacity of the sow. However, these molecular regulation mechanisms affecting sow litter size are still unclear. In this study, abundant lncRNAs were differentially expressed in the SLS and LLS ovary tissue, revealing lncRNAs to have specific roles in ovarian function but not by-product of mRNA. Moreover, an mRNA-miRNA-lncRNA network was constructed according to the competitive endogenous RNA mechanism. The lncRNA LOC100513133 and LOC102168075 could be as ceRNAs containing potential binding sites for miR-26b, let-7g and miR-125b (Cao, et al. 2015b; Zhang, et al. 2020b). Previous studies have shown miR-125b is potential regulatory factor for the development of polycystic ovarian syndrome and miR-26b has a protective effect on ovarian granulosa cells (Xuan et al., 2024; Liu S. et al., 2020). Therefore, our next step is to explore the role of lncRNA in pig ovarian function.

## Conclusion

This study is to map the expression of mRNA and lncRNA expression in porcine ovaries with smaller and larger litter size. Thousands of lncRNAs were identified, and several of them showed very different abundances in ovary tissues. We further constructed ceRNA network, and found that LOC100513133 and LOC102168075 might act as ceRNA. Our research has increased the understanding of the genetic mechanism of pig ovarian function and may provide new molecular target for the precise breeding of pig in China.

## Data Availability

The RNA-seq data of porcine ovary have been uploaded to the GEO database of NCBI and the data ID is GSE136592.

## Supplementary material

SUPPLEMENTARY FIGURE S1 mRNA-miRNA-lncRNA interaction network. The green circle represents the downregulated mRNA, the red circle represents the upregulated mRNA, the blue V-shape represents the downregulated miRNA, the pink V-shape represents the upregulated miRNA, the purple diamond represents the downregulated lncRNA, and the orange diamond represents the upregulated lncRNA.
