# Supplementary figures and images for: Discovery of differentially expressed lncRNAs in porcine ovaries with smaller and bigger litter size
Source: Front Genet. 2025 Apr 16;16:1498076. doi: 10.3389/fgene.2025.1498076 (PMC12040972; doi:10.3389/fgene.2025.1498076)

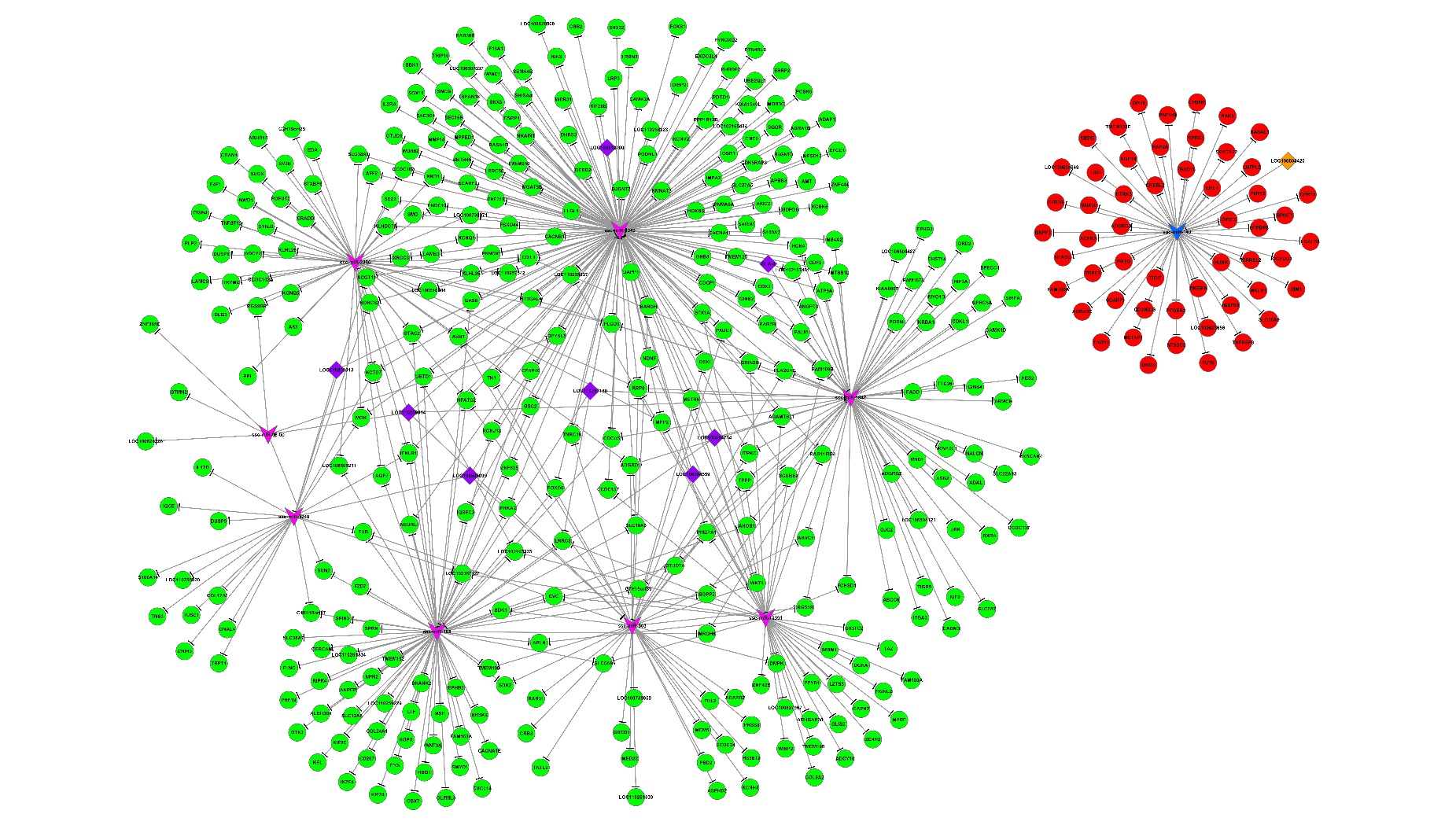

Supplement: Supplementary file 2 [file Image1.jpeg]
