# Supplementary material for: Discovery of differentially expressed lncRNAs in porcine ovaries with smaller and bigger litter size
Source: Front Genet. 2025 Apr 16;16:1498076. doi: 10.3389/fgene.2025.1498076 (PMC12040972; doi:10.3389/fgene.2025.1498076)
Supplement: Supplementary file 6 [file Table1.doc]

**TABLE S1∣**The primers for qPCR.

| **Name** | **Sequence (5′→3′)** |
| --- | --- |
| EPCAM | F: TGCTCTTTGAATGCGCTTGG |
|  | R: AGAGCCCATCGTTGTTCTGG |
| ERO1β | F: TCACCGGAGTCCTGGATGAT |
|  | R: GAAAGGACAGGGTCGCTTCA |
| LOC100525099 | F: GGAGCCCAGGTTCGATGAAA |
|  | R: AGAGGGTCAGACGTTCGGTA |
| HSD17β2 | F: CCAAACAAACATCGCAGGCA |
|  | R: TAGTCCTGGCCGTAGTCCTC |
| LRP8 | F: GACTGCAAGGACAAGTCGGA |
|  | R: CGTTGTTGTGCAGACACTCG |
| LRP2 | F: ATCAGGGATGGAGAGTGGCT |
|  | R: GGTCGTAGTAGACCCCACCT |
| ACTG2 | F: GAGCGGAAGTACTCGGTCTG |
|  | R: TCCTGTGGACAATGGAAGGC |
| SLIT1 | F: CCAAGTGCCCAGGGATCAAT |
|  | GTGCAAGGCGTGGAAACAAT |
| LOC106506002 | F: AGTGAGTGGAATGAGGTGGG  R: CTCGGTCTCTCAGGGCTATG |
| LOC110256292 | F: AAAACTGAAGTGCACACCCC  R: ATGGCAGACACAGGATTGGA |
| LOC110256263 | F: GCATAGACCCTGACCCTTCA  R: AGACTTCTCAGCCCATCCAC |
| LOC100621695 | F: GTGTGAAGCCCAAGTTCCAG  R: GTCAGACTTAGGGCAGGGTT |
| LOC110255514 | F: CACCAGCAGACAGAAGAGGA  R: GCAGGACGTAGATACTCCCC |
| LOC100621235 | F: GCTGGAAAAGGTGAGGAAGC  R: TCCTGGGCTCAAATCAAGGT |
| LOC102168075 | F: TGTCTTTCCGCCTCACTTCT  R: AGAACACAACTGCAAACGCT |
| LOC110257884 | F: AGCAAGCCCATCAAACACAG  R: TATGTATGTACGGCGGGCTT |
| LOC110262147 | F: TGACATCTGAATCTCCGCGA  R: TGCCCAAGAGAAGCTGTACA |
| LOC110256037 | F: CCCAATCCACTTTTGCCGAA  R: CTTGAAGCCCAGCATCCATC |
| LOC110258382 | F: AAGCAGGGTTGAGTGAGGAA  R: AGAGAAAGTCCACAGGGCTC |
| LOC106510454 | F: CACTGTCCTCCTCCTCGATC  R: TACAAGTCAAGGAGCCAGGG |
| β-actin | F: GGACTTCGAGCAGGAGATGG  R: AGGAAGGAGGGCTGGAAGAG |
