# Supplementary material for: Discovery of differentially expressed lncRNAs in porcine ovaries with smaller and bigger litter size
Source: Front Genet. 2025 Apr 16;16:1498076. doi: 10.3389/fgene.2025.1498076 (PMC12040972; doi:10.3389/fgene.2025.1498076)
Supplement: Supplementary file 8 [file Table2.doc]

Table S2 Prolificacy characteristics of LLS and SLS pigs

| Group | Sample name | Production times | Age  (months) | Number of offspring（M±SE） |
| --- | --- | --- | --- | --- |
| LLS | LLS1 | 4 | 30 | 15.75±1.31 |
|  | LLS2 | 4 | 31 | 16.75±1.11 |
|  | LLS3 | 4 | 30 | 16.25±1.75 |
|  | LLS4 | 4 | 32 | 16.00±1.22 |
| SLS | SLS1 | 4 | 31 | 7.75±1.80 |
|  | SLS2 | 4 | 30 | 8.50±1.66 |
|  | SLS3 | 4 | 30 | 9.00±1.96 |
|  | SLS4 | 4 | 33 | 6.50±2.40 |
